# Expression of genes associated with carbohydrate metabolism in cotton stems and roots

**DOI:** 10.1186/1471-2229-9-11

**Published:** 2009-01-22

**Authors:** Earl W Taliercio, Gabriela Romano, Jodi Scheffler, Brian G Ayre

**Affiliations:** 1 USDA/ARS, 3127 Ligon St., Raleigh, North Carolina 27695, USA; 2 USDA/ARS, 141 Experiment Station Road, Stoneville, Mississippi 38776, USA; 3 University of North Texas, Department of Biological Sciences, 1504 W. Mulberry, SRB Rm 120, P.O. Box 305220, Denton, TX 76203 5220, USA

## Abstract

**Background:**

Cotton (*Gossypium hirsutum *L) is an important crop worldwide that provides fiber for the textile industry. Cotton is a perennial plant that stores starch in stems and roots to provide carbohydrates for growth in subsequent seasons. Domesticated cotton makes these reserves available to developing seeds which impacts seed yield. The goals of these analyses were to identify genes and physiological pathways that establish cotton stems and roots as physiological sinks and investigate the role these pathways play in cotton development during seed set.

**Results:**

Analysis of field-grown cotton plants indicated that starch levels peaked about the time of first anthesis and then declined similar to reports in greenhouse-grown cotton plants. Starch accumulated along the length of the stem and the shape and size of the starch grains from stems were easily distinguished from transient starch. Microarray analyses compared gene expression in tissues containing low levels of starch with tissues rapidly accumulating starch. Statistical analysis of differentially expressed genes indicated increased expression among genes associated with starch synthesis, starch degradation, hexose metabolism, raffinose synthesis and trehalose synthesis. The anticipated changes in these sugars were largely confirmed by measuring soluble sugars in selected tissues.

**Conclusion:**

In domesticated cotton starch stored prior to flowering was available to support seed production. Starch accumulation observed in young field-grown plants was not observed in greenhouse grown plants. A suite of genes associated with starch biosynthesis was identified. The pathway for starch utilization after flowering was associated with an increase in expression of a glucan water dikinase gene as has been implicated in utilization of transient starch. Changes in raffinose levels and levels of expression of genes controlling trehalose and raffinose biosynthesis were also observed in vegetative cotton tissues as plants age.

## Background

Cotton (*Gossypium hirsutum *L) is an important source of fiber for the textile industry. Cotton is a perennial plant that is grown as an annual row crop in much of the world. As a perennial, cotton plants naturally make provisions for growth in the next season by storing starch in stems and roots. Availability of stored starch to support seed and fiber development may impact yield. Additionally, fibers are elongated ovular trichomes that are sites of cellulose deposition which also require photoassimilate from leaves to provide the glucose (GLC) subunits to make cellulose [1]. At peak productivity, leaves subtending developing bolls export up to 33% of photoassimilate to vegetative parts of the plant and up to 28% of carbon needed to complete boll maturation comes from previously assimilated sources [2]. At the whole plant level a large portion of reproductive development occurs after canopy photosynthesis declines [2]. In fact, part of the photosynthetic apparatus appears to be sacrificed to provide nitrogen for developing bolls [3].

There are no annual species of cotton to help breed truly annual cotton cultivars although current cultivars have been "annualized" to make them better adapted for agriculture. For example flowering of wild cotton is sensitive to day length but flowering of cultivated cotton is largely insensitive to day length. In studies of greenhouse grown cotton plants, levels of starch in stems and roots of cultivated cotton peaked at about 90 days post emergence then dropped [4]. Levels of starch in stems and roots of wild cotton remained high throughout the same period of development. One interpretation of the difference in starch levels between cultivated and wild cotton is that part of the "annualization" of cultivated cotton involved the redistribution of a portion of stored starch to reproductive sinks. Similar levels of starch stored in stems and roots of antique and modern cotton cultivars indicated that redistribution of these reserves may not be subject to selection in breeding programs and therefore not optimized for crop production [5]. Cotton plants produce many more flowers than develop into productive bolls demonstrating that cotton plants have untapped potential to set more bolls [6,7]. It would be desirable to redirect photoassimilate from unproductive starch reserves to boll development because stored reserves not made available to reproductive tissues are wasted when cotton is grown as an annual crop.

Previous statistical analyses of expressed sequenced tags (ESTs) isolated from cotton stems identified increased expressions of genes associated with lignin and starch biosynthesis consistent with starch and lignin production in the tissues investigated [8]. Our goal was to identify genes and physiological pathways that establish cotton stems and roots as physiological sinks and investigate the role of these pathways in cotton development during seed set. We compare the expression of over 11,000 genes between stems and roots before and after they begin to store starch. Expression data are validated using quantitative-PCR (qPCR) and the physiological impact of altered gene expression is largely confirmed by measuring carbohydrate levels during relevant period of development.

## Results

### Starch analysis

**Figure 1:**
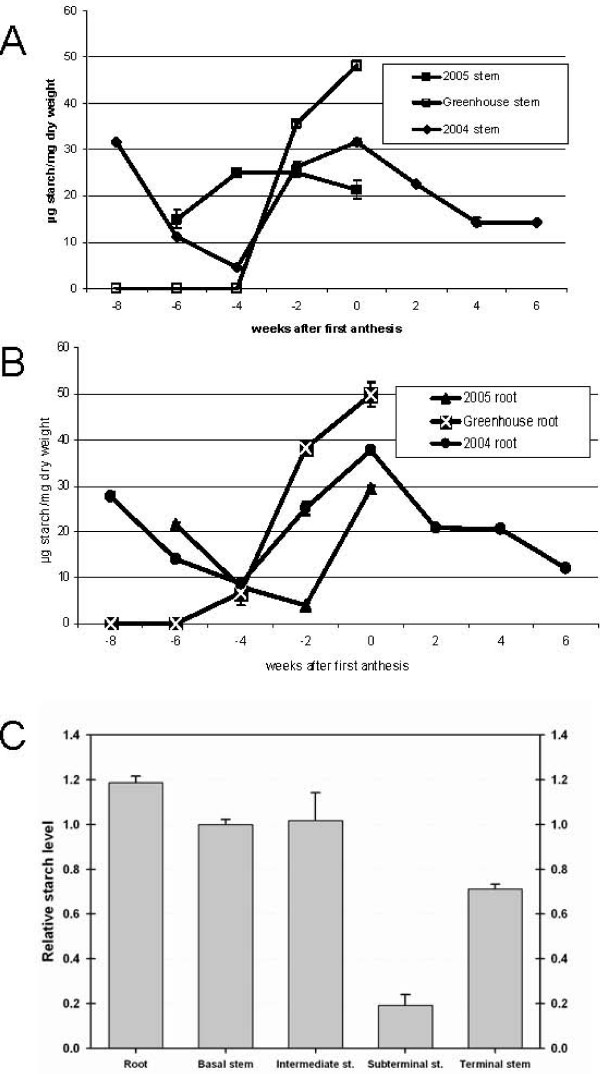
**Starch in cotton stems and roots**. Time 0 indicates plants with the first open flower. Negative numbers indicate weeks prior to flowering, positive numbers indicate weeks after anthesis of the first flower. Panel A. The amount of starch determined for basal stems harvested as indicated. Panel B The amount of starch measured in roots from cotton plants harvested as indicated. Panel C. The relative amount of starch was measured in 0 week plants grown in the field in 2004. The bars indicate standard error.

Roots and basal stems were harvested at two-week intervals from field-grown plants in 2004 and 2005 and greenhouse-grown plants in the winter of 2004. The ground samples were dried and the starch levels determined. Fig. 1A shows the amounts of starch in stems and Fig. 1B the amount of starch in roots over the period harvested with starch levels at first anthesis grouped at time "0". There was a peak in starch approximately coincident with anthesis of the first flower where starch accounted for about 5% of the dry weight of the field-grown sample at peak levels. In field-grown plants harvested in 2004, starch was significantly reduced two weeks after flowering and continued to trend downward but still constituted ~1.5 % of dry weight after six weeks. We noted an early peak in starch levels in field-grown samples accounting for up to 3% of dry weight that was not observed in greenhouse-grown plants.

Roots had higher amounts of starch than basal stems, therefore we wanted to determine if there was a gradient of starch accumulation along the main stem and root. Starch accumulation in plants was evaluated at first anthesis (Fig. 1C) in field-grown plants from 2004. The main stem was divided into four sections and the level of starch determined. Starch levels were high in each section except the subterminal region that included the elongating zone. The terminal section near the shoot apex was also high in starch. Because the terminal stems narrow, they contain a higher proportion of green tissue and associated transient starch. Therefore starch observed in the terminal section could be transient starch instead of stored-starch. Terminal sections stained with iodine indicated starch was associated with nonphotosynthetic tissues (data not shown). Additionally, starch grains from roots and storage tissues visualized under the microscope consistently showed differences in morphology. Starch grains were measured to determine if the size of starch grains in the upper stem were comparable in size to starch stored in other parts of the stem and roots or if it was more similar to transient starch in leaves. The average size of the starch grains from upper stem was more similar in size to the grains from the root of the same plants than starch from the leaves (p < 0.05, Table 1). In stems, starch grains substantially increased in size between -2 weeks and 0 weeks in the greenhouse-grown samples (p < 0.05). Starch grains from leaves appeared elongated whereas starch grains from the stems and roots were round. Starch grain diameters were measured along the perpendicular axis and the ratios determined to validate this observation. The ratios of ~0.7 and ~0.95 (short*long^-1 ^axis, p < 0.05) in starch grains from leaves and stems, respectively, confirmed that transient starch grains from leaves were more ovoid in leaves of this genotype. Taken together these data indicated that the terminal section of cotton stems store starch and there was not a gradient of starch accumulation along the stem, particularly below the terminal stem.

**Table 1:** Starch Measurements

**Tissue**	**Age**	**Growth**	**Diameter (std.err)**
root	0 week	field 2004	6.69 (0.180) **b**
root	6 week	field 2004	5.29 (0.166) **c**
terminal stem	0 week	field 2004	5.77 (0.174) **c**
basal stem	-2 week	greenhouse	4.53 (0.192) **d**
basal stem	0 week	greenhouse	8.26 (0.201) **a**
leaf	0 week	field 2004	3.62 (0.158) **e**

Diameter of starch granules (μm) from leaves (long axis), stems and roots of cotton. The samples are labeled as described in figure 1. Means followed by the different letters are significantly different (p < 0.05).

### Microarray analysis of RNA from cotton stems and roots

RNA was isolated from roots and stems representing stages in development that were low in starch or stages in development that were rapidly accumulating starch. Probes derived from these RNAs were hybridized to three microarrays. On the first microarray genes expressed in -4 W and -2 W field-grown lower stems from 2004 were compared, on the second microarray genes expressed in -4 W and -2 W greenhouse-grown lower stems were compared and on the last microarray genes expressed in -4 W and -2 W greenhouse-grown roots were compared. Selected genes that were differentially expressed are shown in Table 2 and a complete set of data is available from GEO. No mRNAs were identified that were specifically up-regulated at the two-fold level in low starch samples relative to starch-accumulating samples. Examination of the original information pertaining to the array did not indicate an error labeling the RNA nor were all of the arrays processed at the same time. The genes represented on this array were primarily selected from unique ESTs derived from fiber initials, stems and roots [9]. It is likely that stages of development representing low starch samples were underrepresented on the microarray introducing a bias against genes up-regulated in these tissues.

**Table 2:** Differentially Expressed Genes

Name	R	R_Probt	GenBankInfo	Expect	localization
Contig7205	-6.02	0.0035	UDPglucose 4-epimerase-like protein	e-158	N
Contig2425	-5.55	0.0011	starch branching enzyme I	e-131	Y
Contig4287	-5.37	0.0015	granule-bound starch synthase Ib precursor	9e-99	Y
Contig4122	-5.06	0.0067	1802404A starch phosphorylase	e-103	N
Contig12082	-4.91	0.0123	ADP-glucose pyrophosphorylase large subunit	e-123	N
Contig14936	-4.88	0.0016	starch branching enzyme	8e-07	Y
Contig5410	-4.81	0.0030	putative raffinose synthase	e-149	NA
Contig4985	-4.80	0.0012	phosphoglucomutase	8e-91	NA
Contig3321	-4.73	0.0009	starch synthase II precursor	7e-63	Y
Contig16866	-4.46	0.0030	trehalose-6-phosphate synthase	8e-70	NA
Contig16	-4.39	0.0067	starch phosphorylase	1e-42	Y
Contig6949	-3.91	0.0116	trehalose-6-phosphate phosphatase, putative	e-145	NA
Contig3887	-3.46	0.0112	putative sucrose transporter [Vitis vinifera]	3e-60	NA
Contig3	-3.42	0.0042	glucose-6-phosphate isomerase	2e-07	NA
Contig6516	-3.36	0.0057	alpha-amylase	e-114	N
Contig4765	-3.33	0.0040	raffinose synthase	9e-32	NA
Contig598	-3.30	0.0188	putative phosphoglucomutase	2e-67	NA
Contig13568	-3.13	0.0183	putative sugar transporter	5e-86	N
TMIRS_132_G09.F	-3.09	0.0048	galactinol synthase	3e-14	NA
Contig14868	-3.08	0.0009	Disproportionating enzyme	2e-91	Y
Contig5239	-3.07	0.0071	sucrose phosphate phosphatase	2e-36	NA
Contig15191	-3.01	0.0101	hexokinase	7e-49	NA
Contig4991	-2.77	0.0044	putative trehalose 6-phosphate synthase	5e-51	NA
TMIRS_163_E04.F	-2.70	0.0128	putative trehalose-6-phosphate phosphatase	1e-41	NA
Contig15847	-2.68	0.0072	trehalose-6-phosphate synthase	1e-18	NA
Contig6597	-2.65	0.0107	hexokinase 2	1e-29	NA
Contig4561	-2.63	0.0040	putative trehalose-6-phosphate synthase	0.0	NA
Contig15338	-2.55	0.0088	putative hexose transporter protein	1e-81	NA
Contig17843	-2.37	0.0201	hexokinase	1e-17	NA
Contig16302	-2.37	0.0221	glucan water dikinase (Starch-related R1 protein)	e-118	Y
Contig1339	-2.24	0.0015	starch synthase II-2 precursor	e-134	Y
Contig2930	-1.20	0.6448	ribosomal protein S3	2e-12	NA
Contig16061	-1.06	0.8759	expansin-1	e-131	NA

Genes differentially expressed in starch accumulating cotton stems and roots. This is a partial list of genes that are increased in expression in starch accumulating stems and roots. Sequences refers to the designation in the original assembly, R is the ratio of the signal from low starch/high starch channels, the negative reciprocal was used if the ratio was less than 0, therefore more negative numbers are more highly expressed in starch accumulating tissues. R_probt is the statistical support for R. GenBankInfo is a brief description of the gene and E is the expect value from GenBank. Localization indicates if the best cognate is likely to be localized to the plastid. "Y" indicated the cognate is likely to be localized to the plastid, "N" means it is not and "NA" means it was not evaluated for localization.

**Figure 2:**
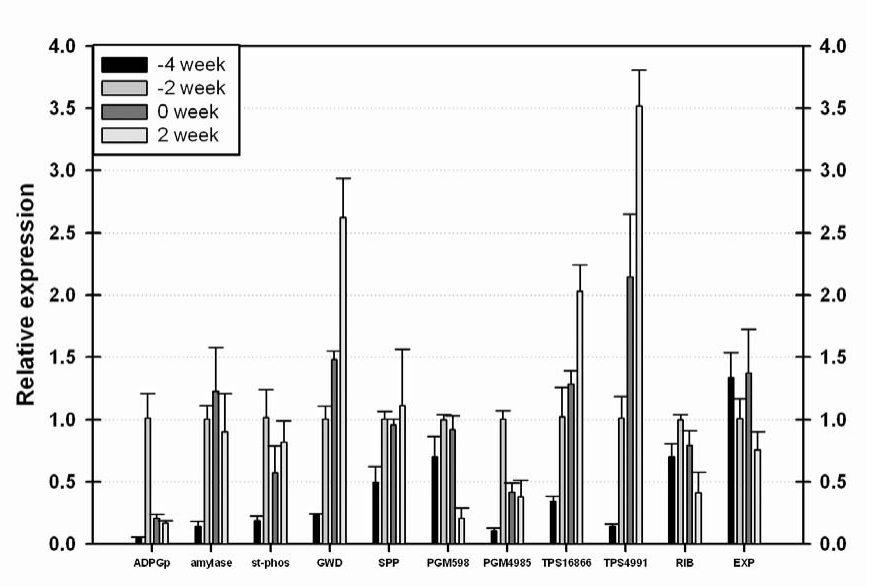
**QPCR of selected genes**. QPCR confirmed the differential expression of seven transcripts putatively encoding an ADPGp, an α-amylase, a starch phosphorylase (st-phos), GWD, a phosphoglucomutases (PGM 4985) and two trehalose phosphate syntases (TSP 16866 and TSP 4991) in field-grown cotton roots. The SPP fell just short of the two fold cut-off for differential expression. One PGM (PGM598) identified as differentially expressed on microarrays was not differentially expressed in these root samples between -4 week and -2 week. QPCR also confirmed the relatively constant expression (less than two fold variation) predicted by microarray analysis of a ribosomal protein gene (RIB) and an expansin gene (EXP). The RNAs analyzed were from cotton root harvested at the indicated times.

The microarray data were validated using qPCR to confirm differential levels of selected transcripts in -4 W (low starch) and -2 W (accumulating starch) and extended to 0 W and 2 W field-grown roots from 2004 (Fig. 2). Differential expression was confirmed for 7 genes with differential expression ranging from 2.7 to 4.9 on the microarray using qPCR. An eighth gene sucrose phosphate phosphatase (SPP) gene fell just below the two-fold cut-off used to designate differential expression. One phosphoglucomutase (PGM598) identified as differentially expressed on the microarray was not differentially expressed in these roots between -4 W and -2 W but decreased in expression later in development (Fig. 2). An expansin gene and a ribosomal protein expressed in about equal levels among the samples investigated, according to the microarray data, were also evaluated with qPCR. These genes showed no differential expression as expected. Previous analysis of ESTs derived from cotton stems indicated expression of genes associated with lignin biosynthesis increased as stems matured [8]. At least 25 genes associated with lignin biosynthesis increased in expression in the more mature stems and roots as expected. Multiple genes associated with ethylene signaling, auxin signaling, jasmonate signaling and salicylic acid response were also differentially expressed. Detailed information is available at GEO. Statistical analyses of differentially expressed genes were performed at GO stat as previously described [9,10]. These analyses indicated genes associated with "carbohydrate metabolism" (GO:0044262, p = 0.023) were increased in expression as would be expected for starch accumulating tissues. Genes associated with "transcriptional activity" (GO:0003700, p = 0.009) are also increased in expression in starch accumulating tissues.

### Expression of genes associated with starch metabolism

Many genes associated with starch biosynthesis were up-regulated as starch accumulated, consistent with the starch increase observed in these tissues (Table 2). These include ADPglucose-pyrophosphorylase (ADPGp, EC .2.7.7.27), a variety of starch synthases and starch branching enzymes. Quantitative PCR confirmed a nearly ten-fold increase in the level of transcript encoding the large subunit of ADPGp between -4 W and -2 W and a subsequent significant drop in expression in cotton roots (Fig. 2). We wanted to determine the probable subcellular localization of some of the genes associated with starch metabolism but many of the gene models represented on this microarray lacked completed coding regions. Therefore the best annotated cognate for which a complete coding strand was available was analyzed *in-silico *for localization to the plastid (Table 2). Most had a high probability of plastid localization. The exceptions were the ADPGp large subunit and one of the starch phosphorylases.

### Analysis of sucrose (SUC), GLC and fructose (FRC) metabolism

SUC is used to transport carbon from source tissues to the sink tissues in most plants. Multiple sugar transporters appear to be differentially regulated in starch accumulating tissues (Table 2). Multiple invertases that cleave SUC to form GLC and FRC and an invertase inhibitor are up-regulated in starch accumulating tissues. The only enzyme that synthesizes SUC, sucrose phosphate synthase did not appear to be differentially regulated. An increase in an SPP transcript that appeared to be differentially expressed on the microarrays fell just below the two-fold cut-off for designating a gene as differentially expressed using qPCR (Fig. 2).

Analysis of expression of genes encoding GLC metabolizing enzymes identified several relevant genes that increased in expression in starch accumulating stems and roots. Multiple genes encoding phosphoglucomutase (EC .5.4.2.2) increased in expression in starch-storing stems (Fig. 2). This pattern of expression was confirmed for one of the genes by qPCR. The qPCR analysis also indicated that the mRNA level of two of these genes dropped in later stages of stem development.

Levels of soluble carbohydrates were measured in relevant stem and root samples to gain insight into the role these sugars play in starch storing tissues (Table 3). Hexose levels in cotton stems and roots increased during stages of starch degradation. The relative level of FRC compared to GLC/galactose (GAL) in particular increased. There was also a peak in SUC that correlated with the peak in FRC. The relative levels of various soluble carbohydrates were consistent between stems and roots harvested the same year.

**Table 3:** Measurement of Soluble Sugars

	**SUC**	**GLC/GAL**	**FRC**	**GOL**	**RAF**
0 week stem	29.60 (1.10)	24.77 (0.98)	6.44 (0.27)	nd	0.11 (0.00)
2 week stem	34.14 (2.28)	22.21 (1.49)	9.53 (0.68)	nd	0.19 (0.02)
4 week stem	40.82 (3.11)	28.26 (2.32)	14.95 (1.24)	nd	0.69 (0.07)
6 week stem	37.94 (1.96)	21.23 (1.63)	11.33 (0.97)	nd	0.04 (0.00)
-2 week root	49.33 (1.65)	21.62 (0.56)	5.57 (0.11)	0.15 (0.01)	0.11 (0.01)
0 week root	50.92 (2.59)	20.76 (1.72)	4.95 (0.47)	0.16 (0.02)	0.12 (0.02)
2 week root	44.39 (1.53)	21.69 (0.76)	7.48 (0.69)	0.38 (0.01)	0.16 (0.01)
4 week root	56.16 (2.21)	33.76 (1.25)	15.51 (0.52)	nd	0.90 (0.04)
6 week root	48.80 (2.13)	27.40 (1.97)	13.17 (1.41)	nd	0.10 (0.01)

Measurements of soluble sugars in cotton stems and roots. Measurements of sucrose (SUC), glucose and galactose (GLC/GAL), fructose (FRC), galactinol (GOL) and raffinose (RAF). The standard error of three replications is included in parentheses and nd, none detected. A replication is an isolation of soluble sugars from 50 mg of samples. Units are nmoles sugar/mg fresh weight of sample.

### Metabolism of other carbohydrates

Genes associated with raffinose (RAF) and trehalose (TRE) biosynthesis increased in starch accumulating tissues (Table 2). Differential expression of two trehalose phosphate synthases (EC .2.4.1.15) was confirmed by qPCR. Trehalose (α-D-glucopyranosyl-1,1-α-D-glucopyranoside) is a disaccharide of two GLC [11]. RAF (α-1,6-galactosyl-SUC) is a GAL linked to a SUC [12,13]. The raffinose family of oligosaccharides (RFO) includes stachyose and verbascose in which 2 or 3 galactose molecules are linked to SUC, respectively. There was an increase in genes potentially participating in the metabolism of RFO, including UDP-glucose/galactose 4-epimerase (EC .5.1.3.2), galactinol synthase (EC .2.4.1.123) and α-galactosidase (EC .3.2.1.22) (Table 2). UDP-glucose/galactose 4-epimerase interconverts UDP-glucose and UDP-galactose and is an important pathway for GAL production. Galactinol synthase synthesizes galactinol from UDP-GAL and inositol. Galactinol is the galactosyl donor for RFO biosynthesis. α-Galactosidases play a role in the hydrolytic degradation of RFO [14,15]. Analysis of soluble carbohydrates in cotton stems and roots supported an increase in raffinose and the increase continued as starch was degraded (Table 3). The levels of trehalose were too low to reliably measure.

## Discussion and Conclusion

We present work that integrates measurements of starch and soluble carbohydrate levels with analyses of gene expression in starch accumulating cotton stems and roots. Consistent with previous reports there is a rapid increase in the levels of starch stored in the stem and root of cotton plants approximately coincident with bloom of the first flower [4]. This high starch content in stems and roots at first anthesis was followed by a subsequent decrease in starch levels in field-grown plants. There was often a novel peak in starch levels prior to first bloom in field-grown plants that was not observed in greenhouse-grown plants.

Roots were generally higher in starch than stems. Analysis of starch in stems at first anthesis failed to demonstrate a clear gradient of starch accumulation along the stem, particularly below the elongation zone. Starch appeared to be synthesized in a concerted fashion along primary stems below the elongating zone of flowering plants. Analysis of starch grains showed that transient starch in leaves could be distinguished from stored starch in stems and roots based on shape and size. Additionally, starch in stems above the elongation zone appeared more similar to stored starch in stems and roots than transient starch in leaves indicating almost the entire stem stores starch.

Microarray analyses were used to investigate changes in gene expression associated with changes in starch levels and were validated using qPCR analyses of expression of selected genes. Statistical analysis of the data indicated an increase in genes associated with carbohydrate metabolism as expected for tissues accumulating starch. Additionally, expression of genes associated with transcription factor activity also increased indicating a potential role for changes in gene expression as stems and roots started to store starch. Some of these transcription factors are likely to play roles in regulating carbohydrate metabolism.

There must be an increased flow of SUC from leaves to stems and roots as starch accumulates. Consistent with this requirement was an increase in expression of genes associated with sugar transport in these tissues. Transcripts encoding invertase and invertase inhibitors increase in tissues accumulating starch. Invertase converts SUC to GLC and FRC providing glucose for starch biosynthesis. We also note that the change of expression of sucrose synthase which converts SUC to GLC-UDP and FRC fell just below the two-fold threshold for consideration. Increases in levels of transcripts encoding hexokinases, phosphoglucomutase and isomerases were consistent with the production of Glucose-1-phosphate for starch biosynthesis (Table 2). SUC and FRC levels peaked in both roots and stems at 4 W post anthesis. Four weeks after first anthesis represents a time of considerable demand for carbohydrates by the developing seeds. Sucrose is synthesized via sucrose phosphate synthase and SPP. Sucrose phosphate synthase synthesizes sucrose-phosphate from GLC-UDP and FRC-P and SPP dephosphorylates sucrose-phosphate. As previously noted the increase in SPP transcripts fell just below the two- fold level in starch accumulating tissues and did not subsequently increase. More detailed analyses of cotton stems after flowering will elucidate important aspects of sucrose metabolism that would allow export of carbon to support seed development.

ADPGp expression is increased in starch storing tissues. ADPGp catalyzes the conversion of glucose-phosphate to glucose-ADP, which is a rate limiting step in starch biosynthesis [16]. As expected, other genes associated with starch biosynthesis such as starch synthase and starch branching enzymes increased in expression during the starch accumulation stage of stem and root development. Starch phosphorylase also appeared to play a role in starch biosynthesis in rice seed [17]. Somewhat unexpected was an increase in expression of genes associated with starch degradation in starch accumulating tissues. QPCR of an α-amylase transcript indicated that these transcript levels increased in starch accumulating tissue and stayed at about the same level as starch decreased. Amylase enzyme activities have been reported to correlate with starch levels in cotton plants [4]. α-Amylase is not required for starch degradation in *Arabidopsis *leaves indicating it may have functions other than starch degradation [18]. Starch degrading genes, such as those encoding starch debranching enzymes, play a role in starch maturation. Expression of a glucan water dikinase (GWD) transcript increased in expression in starch accumulating tissues and continued to increase in expression as starch was utilized. GWD phosphorylates starch and is necessary for degradation of transient starch in *Arabidopsis* leaves [19-21].

Most of the proteins directly involved in starch metabolism appeared to be targeted to the plastid as expected. One exception was the ADPGp large subunit. The gene model used on this microarray included the 5' end of the coding sequence but was not predicted to be targeted to the plastid even though other plant ADPGp large subunits were. Failure to localize this ADPGp to the plastid may indicate an error assembling this gene or might indicate this gene is located in the cytosol. A cytosolic localization has been reported for some ADPGp [22].

Analysis of differentially expressed genes identified an increase in transcripts encoding enzymes for TRE biosynthesis and RAF biosynthesis. Analysis of RAF levels and the expression of genes associated with the biosynthesis of RAF and TRE indicated that they peak in expression in field-grown stems and roots well after starch [11,23,24]. The TRE pathway (especially trehalose-6-phosphate) is associated with control of glycolysis, ABA signaling and starch accumulation in Arabidopsis and has been associated with drought stress in cotton [25]. QPCR confirmed an increase in transcripts encoding trehalose phosphate synthase that continued even after starch levels declined. Therefore TRE may play a role in the starch utilization stage of cotton stem development. RAF accumulates prominently in maturing seeds where it is thought to act as a compatible solute in preparation for seed desiccation and as a storage reserve for post-germinative growth [26]. RAF and RFO are thought to play a role in cold and desiccation tolerance in plants and in some plants are prominent transport sugars [12]. The role RFO is playing in cotton is unclear because even at peak levels, they are below those associated with desiccation tolerance. The increase of putative RFO anabolic and catabolic gene expression may point to transient fluctuations in levels, or rapid flux of carbohydrate through this pathway.

Cotton is an unusual crop because it is a perennial that is often grown as an annual row crop. One method of determining how well cotton has been annualized is to measure reserves stored for subsequent regrowth which are unavailable for seed production and therefore wasted in an annual row crop. Starch accounted for about 1.5 % of the dry weight of stems and roots late in boll development. One goal of this research is to identify genes and pathways that could be modified to direct starch into agronomically valuable fiber yield. For example, modification of ADPGp expression reduced starch metabolism in potato [27]. Starch stored in cotton roots and stems prior to flowering appears to be available to developing bolls so it may be more desirable to retain this starch in stems and roots until after flowering and then mobilize all of the starch to support seed and fiber development. It is also important that the mobilization of carbohydrates from the stems and roots is not limited by environmental factors. Improved mobilization of carbohydrates from stem and root may be accomplished by reducing expression of starch biosynthesis genes after flowering and/or by increasing expression of starch degrading enzymes after flowering. Additionally, altering signaling pathways, for example TRE metabolism or expression of relevant transcription factors, may provide valuable targets to coordinately change starch biosynthesis and degradation in a way that favors increased yield. These approaches could be explored by targeting genes presented in this study using standard methods of cotton transformation.

## Methods

### Plant material

The cotton cultivar STV4793R was grown in Stoneville, MS in 2004 and 2005. The same cultivar was planted in a greenhouse in Stoneville, MS (2004) at two-week intervals and the root and main stems harvested simultaneously. The main stem was divided into approximately four sections of three nodes. The stem sections nearest the root and extending to the apical meristem are termed basal, intermediate, subterminal and terminal stems. Stems and roots were harvested between 8:00 AM and 10:00 AM. Leaves were harvested at 1:00 PM to recover greater levels of starch. Plant material was frozen in liquid nitrogen and ground with liquid nitrogen in a 3 hp Warring Blender (Torrington, CT, USA) at the highest speed for 3 min. Each sample represented at least three plants and 5 g of the ground material were freeze-dried in 50 mL tubes for 5 days. The freeze-dried material was stored at -20°C.

### Starch assays

The method described by Hendrix was used to determine the amount of starch in each sample [28]. Briefly, the soluble sugars were extracted from 50–100 mg of the dried material using 3 extractions with 1 mL hot 80% ethanol. The extracted plant material was pelleted in a microfuge tube and the starch gelatinized by heating to 95–98°C in 0.1 M KOH. The samples were neutralized with 0.2 mL 1 M acetic acid and the pH adjusted to 7 by the addition of acetic acid as needed. The starch was digested with 200 μL (300 units) α-amylase solution at 85°C for 30 min. The pH was lowered to 5 with addition of acetic acid, 1 mL of amyloglucosidase was added (125 units), incubated at 55°C for 60 min and 95–98°C for 4 min. The sample was centrifuged and the supernatant solution brought to 6 mL with water. The α-amylase (Sigma), St. Louis, MO, USA and amyloglucosidase (Sigma) were prepared exactly as described by Hendrix, including testing various amounts of samples to be sure starch digestion was complete. The GLC content of each sample was also determined according to Hendrix [28]. Starch was determined by multiplying the total amount of GLC from each sample by 0.9. Each value represents the analysis of starch from three or four replications of 50–100 mg of dried material from each time point. Statistical analysis was done using EXCEL (Microsoft, Seattle, WA).

### Starch imaging

Starch was isolated using a modification of the method described in Ritte et al [29]. Five grams of ground plant material were added to 25 mL starch extraction buffer (100 mMHEPES pH8, 5 mMDTT, 0.05% triton-X-100) and filtered through a double layer of cheese cloth. The filtrate was filtered through a 30 μm nylon mesh. Analysis of starch isolated without filtration through the mesh indicated that starch grains were much smaller than 30 μm and that this filtration step did not remove larger grains. The filtrate was centrifuged at 1000 × g for 5 min to pellet the starch. The supernatant solution was carefully removed to avoid disturbing the pellet. The pellet was resuspended in 10 mL extraction buffer and carefully layered over 5 mL of a 95% percoll pad (5% extraction buffer, 95% percoll) and centrifuged for 15 min at 2000 × g. The supernatant solution, including the percoll pad was carefully removed and the starch pellet resuspended in 30 mL sterile extraction buffer. Starch was allowed to settle to the bottom of the tube for 18 hrs at 4°C. The supernatant solution was carefully removed and the settled starch was resuspended in 10 mL extraction buffer and centrifuged at 1000 × g for 5 min. The starch pellet was resuspended in 10 mL water and centrifuged for 5 min at 1000 × g three times. The starch pellet was resuspended in 10 mL acetone and centrifuged for 5 min at 1000 × g three times. The pellet was air dried overnight in a chemical hood and stored at -20°C.

About 10 mg of starch were suspended in 50% glycerol and dilutions made to allow best separation of individual grains. The suspensions were stained with I_2_KI (2%KI, 0.2%I_2_) and visualized with an Axiovision camera (Zeiss, Thornwood, NY, USA) using the Axiovision 4.4 software (Zeiss). Starch grain diameters were measured with ImageJ 1.36 [30]. Statistical analyses were performed with SAS (Cary, NC, USA).

### Soluble sugar analyses

Soluble sugars were extracted from 50 mg of frozen samples (fresh weight) using two extraction with five volumes of MCW (methanol:chloroform:water, 12:5:1) including 10 μM lactose as an internal standard. After extraction, water (0.6 volumes) was added to the combined extracts to separate the chloroform and aqueous phase. Samples were centrifuged for 15 min at 13,000 × g and the aqueous layer transferred to a 15 mL tube and lyophilized. The dried extracts were resuspended in 300 μL HPLC grade water. Neutral sugars were isolated from each sample by passing the solution through a column consisting of AG50W cation exchange resin (H+ form; BioRad, Carlsbad, CA, USA), polyvinyl polypyrrolidone (Sigma), and AG1 anion exchange resin (formate form, Bio-Rad); 250 μL, 100 μL and 250 μL bed volumes, respectively (top to bottom), and eluting with 1.7 mL water. Each eluate was filtered through a 0.22 μm nylon HPLC filters (Corning-Costar, Lowell, MA, USA). Sugars were resolved and quantified against standards by high-performance anion exchange chromatography with pulsed-amperometric detection (HPAEC-PAD) using a CarboPac PA20 column at 40°C, 50 mM NaOH eluent, and quadruple waveform, as recommended by the instrument manufacturer (Dionex, Sunnyvale, CA, USA). GLC and GAL co-elute under these conditions. Values were normalized against lactose. A separate experiment confirmed that these samples did not include molecules that interfered with the use of lactose as an internal standard. Calculations and statistical analyses were done using EXCEL.

### RNA extraction and microarray analysis

Total polyribosomal RNA was isolated from the selected tissues by standard methods [31]. The quality of the RNA was confirmed on a bioanalyzer (Agilent, Palo Alto, CA, USA) and probes derived from various RNAs representing tissues with different starch levels were labeled with CY3 or CY5. The labeled nucleic acids were hybridized against 3 microarrays made by Agilent (Palo Alto, CA, USA) that have been described in detail elsewhere [9]. On the first microarray, genes expressed in basal stems 4 weeks and 2 week before anthesis (-4 W and -2 W) grown in the field in 2004 were compared. On the second microarray, genes expressed in -4 W and -2 W greenhouse-grown basal stems were compared, and on the last microarray genes expressed in -4 W and -2 W greenhouse-grown roots were compared. The hybridization and data collection was performed by MoGene (St Louis, MO, USA) using standard methods [9]. These data have been deposited in the NCBI Gene Expression Omnibus (GEO) serial number GSE8973 [32]. Statistical analyses are described in Taliercio and Boykin [9].

### Validation of microarray data

Differential expression of selected transcripts was verified using qPCR. A cDNA derived from 0.5 μg of cotton root RNA was used as a template for the primer combination shown in Table 4. The efficiency of each primer set reported in Table 4 was determined on 4-fold or 10-fold dilutions of a mixture of cDNAs. The relative level of expression compared to samples low in starch was calculated by normalizing against rRNA using standard methods [33]. Differential expression was confirmed on at least two separate cDNA preparations.

**Table 4:** Primer Sequences

**Gene**	contig	**sense **5'-3'	**antisense **5'-3'	**efficiency**
sucrose phosphate phosphatase	5239	TGAAAGGGTGCTATGGAGAC	ACACAACAACATCGCTCATC	1.94
phospho gluco mutase	4985	CCGTGGATGGAAGTGTGG	TGGTTCAAATTGCTCGATGTAG	2.01
phospho gluco mutase	598	CGAGGACGGATCACGATTG	AGAGCAACTTCCACAAGAGG	1.96
ADPglucose pyrophosphorylase	12082	TGGACTGGTGAAGATGGATGG	CCGAGGAGCGTGGTATCAG	2.04
trehalose phosphate synthase	16866	GAACATCTCCTGGTGACAATG	GCAAACACATCCGCTACTG	1.95
trehalose phosphate synthase	4991	CCAGCGGTTGTCCATAGAG	ACCAACACACATCACGAAATC	2.01
amylase	6516	CAGAGGATCATTAATTGGATTG	ATTACACCTGGAGGCTTC	1.96
starch phosphorylase	16	TCAAAAGTGGCGTATTCGGATC	TGGGAAGTCTTTGCCAACAAG	2.04
water dikinase	16302	GCTGAGTTCTGGAATGCCTTG	CCTGGTGCTGAAATATGCTCTC	2.14
ribosomal protein	2930	AGTGCTTCTCTGATTGCTCAAGAC	TCGACCTGAACAACATATACGGATC	1.92
expansin	16061	TTGCTTTGTATTGTTCTGTGGTGTG	ACTTGGGCTGCGGGCTAC	1.87

Primers used for qPCR. Gene is a description of the gene function, contig references expression in table 2, sense is the forward primer, antisense is the reverse primer and efficiency is the efficiency of the primer pair in qPCR.

### *In-Silico *localization to plastid

The conceptual open reading frame (ORF) of the ADPGp large subunit was identified using Vector NTI. The protein database at NCBI was queried using BLASTx with candidates lacking the 5'end of the ORF. The plastid localization of these selected sequences were determined using the Chlorop1.1 prediction server [34].

## Authors' contributions

ET planned and analyzed the microarray experiments and wrote the manuscript. JS and GR planned the field component of this work and did the starch image analyses. BA performed and analyzed the HPLC analysis for soluble sugars.
